# Use of Machine Learning to Compare Disease Risk Scores and Propensity Scores Across Complex Confounding Scenarios: A Simulation Study

**DOI:** 10.1002/pds.70165

**Published:** 2025-06-02

**Authors:** Yuchen Guo, Victoria Y. Strauss, Sara Khalid, Daniel Prieto‐Alhambra

**Affiliations:** ^1^ Centre for Statistics in Medicine, University of Oxford Oxford UK; ^2^ Boehringer‐Ingelheim Pharma GmbH & co., KG Germany; ^3^ Department of Medical Informatics Erasmus University Medical Center Rotterdam the Netherlands

**Keywords:** causal inference, disease risk scores, machine learning, propensity scores, treatment effect

## Abstract

**Purpose:**

The surge of treatments for COVID‐19 in the second quarter of 2020 had a low prevalence of treatment and high outcome risk. Motivated by that, we conducted a simulation study comparing disease risk scores (DRS) and propensity scores (PS) using a range of scenarios with different treatment prevalences and outcome risks.

**Method:**

Four methods were used to estimate PS and DRS: logistic regression (reference method), least absolute shrinkage and selection operator (LASSO), multilayer perceptron (MLP), and XgBoost. Monte Carlo simulations generated data across 25 scenarios varying in treatment prevalence, outcome risk, data complexity, and sample size. Average treatment effects were calculated after matching. Relative bias and average absolute standardized mean difference (ASMD) were reported.

**Result:**

Estimation bias increased as treatment prevalence decreased. DRS showed lower bias than PS when treatment prevalence was below 0.1, especially in nonlinear data. However, DRS did not outperform PS in linear or small sample data. PS had comparable or lower bias than DRS when treatment prevalence was 0.1–0.5. Three machine learning (ML) methods performed similarly, with LASSO and XgBoost outperforming the reference method in some nonlinear scenarios. ASMD results indicated that DRS was less impacted by decreasing treatment prevalence compared to PS.

**Conclusion:**

Under nonlinear data, DRS reduced bias compared to PS in scenarios with low treatment prevalence, while PS was preferable for data with treatment prevalence greater than 0.1, regardless of the outcome risk. ML methods can outperform the logistic regression method for PS and DRS estimation. Both decreasing sample size and adding nonlinearity and nonadditivity in data increased bias for all methods tested.

**Summary**

- This study compared the performance of PS and DRS across different treatment prevalences, with a focus on scenarios involving low treatment prevalence and varying levels of confounding.
- The study analyzed the comparative performance of different ML methods and traditional regression approaches for estimating PS and DRS.
- In low treatment prevalence scenarios, DRS had a lower bias compared to PS. In moderate‐to‐high treatment prevalence settings, PS had better covariate balance (lower ASMD) and had comparable or better bias.
- XgBoost outperformed other methods in nonlinear settings with large sample sizes and low treatment prevalence, while LASSO performed best in linear settings with small sample sizes and common treatment prevalence.
- The results suggest that ML methods may extend the applicability of DRS but highlight the need to account for covariate balance when using DRS.

## 1 Introduction

With the growing availability of routinely collected clinical data, the use of observational data to study associations between treatments and outcomes is increasing [1]. However, a major challenge is addressing confounding [2]. Propensity score (PS) analysis, which estimates the probability of treatment given known confounders, is a widely used method to mitigate confounding through techniques like matching, stratification, and weighting [2, 3].

Disease risk score (DRS) analysis presents an alternative approach, where the DRS for a given individual is the predicted probability of the outcome conditional on the confounders and being unexposed. Let *T* denotes treatment, *Y* denotes outcome, and *X* denotes confounders; PS can be expressed as *P*(*T* = 1|*X*) while DRS is *P*(*Y* = 1|*T* = 0,*X*). PS is a function of treatment, whereas DRS is independent of treatment. This property motivated us to test its performance against PS under different treatment prevalences. Scenarios with low treatment prevalence, such as during the COVID‐19 pandemic where only a small subset of individuals received emerging treatments like antiviral medications, pose unique challenges. Low treatment prevalence can lead to poor PS model performance due to limited overlap between treated and untreated groups. At the same time, high outcome risk in such contexts increases the importance of accurately estimating the risk of the outcome. While PS is well‐known and widely applied, DRS is relatively less widely applied in epidemiological literature. DRS could be potentially advantageous in scenarios with low treatment prevalence, where PS has been shown to underperform [4]. These motivated us to explore the comparative performance of PS and DRS under these conditions, especially using machine learning (ML) methods.

To the best of our knowledge, although studies by Arbogast and Ray and Xu et al. [5, 6] have compared PS and DRS, no study has compared their performance using different ML methods. Prior literature has demonstrated that ML methods can improve the accuracy of PS estimation, particularly in complex datasets with high‐dimensional covariates, nonlinear relationships, and interactions among confounders [7, 8, 9, 10, 11, 12, 13, 14, 15, 16, 17]. These improvements stem from the applicability of ML methods to capture complex data structures that traditional regression models may fail to account for. However, DRS remains underexplored in this context, even though it shares similar methodological foundations with PS. Evaluating the performance of ML methods for DRS is important to determine whether these advantages also apply to outcome prediction models used for confounding adjustment.

Therefore, we aimed to assess the performance of different ML methods for PS and DRS estimation under a range of common real‐world scenarios, namely different treatment prevalence and different outcome risk scenarios in the presence of strong confounding.

## 2 Methods

A Monte Carlo simulation study was conducted. The simulation settings of nonlinearity and nonadditivity in Setoguchi's PS simulation research [15] were followed and adapted for the various scenarios under investigation. All code for this paper can be found at https://github.com/MimimimiGuo/PS_ML.

### 2.1 Data Simulation

We simulated datasets using two strategies: (1) independent covariates with no nonlinearity or nonadditivity with treatment or outcome, and (2) covariates with nonlinear and nonadditive correlations with both treatment and outcome. For the second strategy, 15 two‐way interactions (30% of confounders) and 5 cubic terms (10% of confounders) were included to introduce nonadditivity and nonlinearity. Each dataset, linear and nonlinear, contained 50 confounders affecting both treatment and outcome, 5 instrumental variables affecting only treatment, and 1 binomial risk factor affecting only the outcome. Coefficients for the confounders and interactions were randomly sampled from a uniform distribution between 0 and 1, ensuring a mix of weak and strong associations across variables. The true treatment effect was defined as ln(2). Treatment allocation (0 or 1) was simulated using logistic regression, with treatment, 50 confounders, and one risk factor as predictors. Details are provided in Table 1.

**TABLE 1:** Simulation settings.

Covariates categories	Scenario 1–10 (Linear)	Scenario 11–25 (Nonlinear)
Standard normal confounders	$X_{1}$, …, $X_{25}$	$X_{1}$, …, $X_{25}$
Binomial confounders	$X_{26}$, …, $X_{50}$	$X_{26}$, …, $X_{50}$
Nonadditivity terms between normal distribution and binomial distribution	—	*X*6 * *X*26, *X*7 * *X*27, …, *X*10 * *X*30
Nonadditivity terms between normal distribution and binomial distribution	—	*X*11 * *X*12, *X*13 * *X*14, …, *X*19 * *X*20
Nonlinear terms: cubic terms		$X_{1}^{3},X_{2}^{3},\ldots ,X_{5}^{3}$
Nonadditivity terms between cubic terms and binomial distribution	—	$X_{1}^{3}*X_{31},X_{4}^{3}*\;X_{34},\ldots ,X_{5}^{3}*X_{35}$
Standard normal instrumental variables	*X*66, …, *X*70	*X*66, …, *X*70
Binomial risk factor	*X*_71_	*X*_71_

We tested 25 different scenarios, as outlined in Table 2, with varying treatment prevalence, outcome risk percentages, sample size, and data complexity. These scenarios included settings with “strong confounding” and “large sample sizes.” All scenarios involved strong confounding, with multiple confounders affecting both treatment and outcome. However, scenarios classified as nonlinear (Scenarios 11–25) introduced additional complexity through nonlinear and nonadditive relationships, particularly via interaction terms and cubic transformations. These features created more complex structures between covariates, treatment, and outcome compared to the linear scenarios (Scenarios 1–10). Data were generated 100 times for each scenario, which is fewer than the 1000 iterations commonly used in simulation studies [11, 15]. Given the increased number of confounders and the complexity of hyperparameter tuning, we chose 100 iterations to reduce computational cost.

**TABLE 2:** Simulation scenarios.

Scenarios	Treatment prevalence	Sample size	Data structure	Outcome risk
1–5	0.01, 0.05, 0.1, 0.25, 0.5	500	Linear	0.5
6–10	0.01, 0.05, 0.1, 0.25, 0.5	10 000	Linear	0.5
11–15	0.01, 0.05, 0.1, 0.25, 0.5	500	Nonlinear	0.5
16–20	0.01, 0.05, 0.1, 0.25, 0.5	10 000	Nonlinear	0.5
21–25	0.01, 0.05, 0.1, 0.25, 0.5	10 000	Nonlinear	0.02

### 2.2 Propensity Score Estimation

Many PS methods have been evaluated in various studies to mitigate confounding effects [2, 3]. Typically, PS is calculated via logistic regression, incorporating pre‐identified confounders. In the current study, ML methods such as least absolute shrinkage and selection operator (LASSO), XgBoost, and MLP were employed to estimate PS. The treatment status was set as the target variable, and all available covariates were inputted into the ML models. After PS estimation, a matched cohort was created using one‐to‐one nearest neighbor matching without replacement, with a caliper of 0.2 standard deviations of the logit of the PS to improve covariate balance.

### 2.3 Disease Risk Score Estimation

DRS was first introduced by Miettinen in 1976 [18]. It represents the probability of observing a potential outcome conditional on confounders, estimated under the condition that individuals are untreated. There are different approaches to calculating DRS, with two main methods used in previous research by Arbogast and Ray: one based on the unexposed population and the other on the full cohort [5]. For the full cohort, DRS is obtained by regressing the outcome on covariates and treatment across the entire study population (*Y* ∼ *X, T*). The fitted values *P*(*Y* = 1|*T* = 0, *X*) are calculated for the full population by setting the treatment status to unexposed (*T* = 0). For the unexposed population, DRS is calculated by initially regressing the outcome on covariates (*Y* ∼ *X*) for the unexposed individuals, then applying this model to the entire population, with the fitted values *P*(*Y* = 1|*X*) representing the unexposed DRS.

The full cohort DRS has been shown to reduce bias more effectively compared to the unexposed DRS when estimating treatment effects [5] and was therefore used in this study. After DRS estimation, as well as PS, a matched cohort was created using a 1:1 ratio through greedy nearest neighbor matching without replacement, with a caliper of 0.2 standard deviations of the logit of the DRS.

### 2.4 Machine Learning Methods

To estimate PS and DRS, logistic regression was first applied using all simulated confounders. This method incorporated all confounders without additional data‐driven selection steps, mimicking the pre‐selection of confounders from covariates. This approach was designated as the “reference method”. However, this method does not incorporate nonlinearity and nonadditivity.

To address these limitations, we included three widely used supervised ML methods: LASSO, multilayer perceptron (MLP), and eXtreme Gradient Boosting (XgBoost).

LASSO was chosen for its ability to perform variable selection and handle high‐dimensional data efficiently. MLP, a type of neural network, was selected for its ability to model complex, nonlinear relationships. XgBoost, a gradient‐boosting framework, was selected for its strong performance on structured data and its ability to capture nonlinear effects. All ML models underwent hyperparameter tuning using 10‐fold cross‐validation to ensure optimal performance (details available at https://github.com/MimimimiGuo/PS_ML).

### 2.5 Estimands and Metrics

The estimation of PS and DRS using ML methods was conducted using models optimized by hyperparameter tuning. The Brier score loss was selected as the scoring metric for its reliability in measuring the mean squared difference between the predicted probabilities and the actual outcomes, as proposed by Glenn W. Brier [19]. It assesses model calibration effectively. Among various metrics tested, including AUC and calibration slope, the Brier score consistently demonstrated the most reliable performance in estimating final bias, as verified in a previous study [20]. For the LASSO model, the shrinkage parameter was fine‐tuned using a grid search within a 10‐fold cross‐validation framework. Other ML models involved tuning additional hyperparameters. Comprehensive details on hyperparameter tuning and Brier score loss results across all scenarios are documented in the Supplementary Information.

The estimated treatment effect within the matched cohort was determined using logistic regression, with treatment as the sole predictor variable. The true treatment effect was predefined as *ln*(2).

Covariate balance post‐matching was assessed using the average absolute standardized mean difference (ASMD). The standardized mean difference (SMD) for each continuous covariate *X*_*i*_ is calculated as:

$${\mathrm{SMD}}_{i}=\frac{{\bar{X}}_{T,i}-{\bar{X}}_{C,i}}{\sqrt{\frac{S_{T,i}^{2}+S_{C,i}^{2}\;}{2}}}$$

where ${\bar{X}}_{T,i}$ and ${\bar{X}}_{C,i}$ are the means, and *S*_*T,i*_^2^ and *S*_*C,i*_^2^ are the variances of covariate *X*_*i*_ in the treated and control groups, respectively. For categorical and binary variables, the SMD is calculated using proportions:

$${\mathrm{SMD}}_{i}=\frac{P_{T,i}-P_{C,i}}{\sqrt{\frac{P_{T,i}\cdot \left(1-P_{T,i}\right)+P_{C,i}\left(1-P_{C,i}\right)\;}{2}}}$$

where *p*_*T,i*_ and *p*_*C,i*_ are the proportions of the categorical or binary variable in the treated and control groups, respectively. The absolute values of SMD are then calculated, *ASMD*_*i*_ = |*SMD*_*i*_|. The average ASMD is subsequently derived as:

$$\text{ASMD}=\frac{1}{p}\sum_{i=1}^{p}{\text{ASMD}}_{i}$$

where *p* denotes the number of covariates. Lower ASMD values indicate a better balance of covariates after matching.

For evaluating the accuracy of treatment effect estimation, the relative bias was computed as follows:

$$\frac{|\text{Estimated treatment effect}-\text{True treatment effect}|}{\text{True treatment effect}}$$

We reported 95% confidence intervals for all metrics, calculated from the sample mean and standard deviation across 100 simulation rounds for each scenario.

## 3 Results

### 3.1 Comparison Between PS and DRS

**FIGURE 1:**
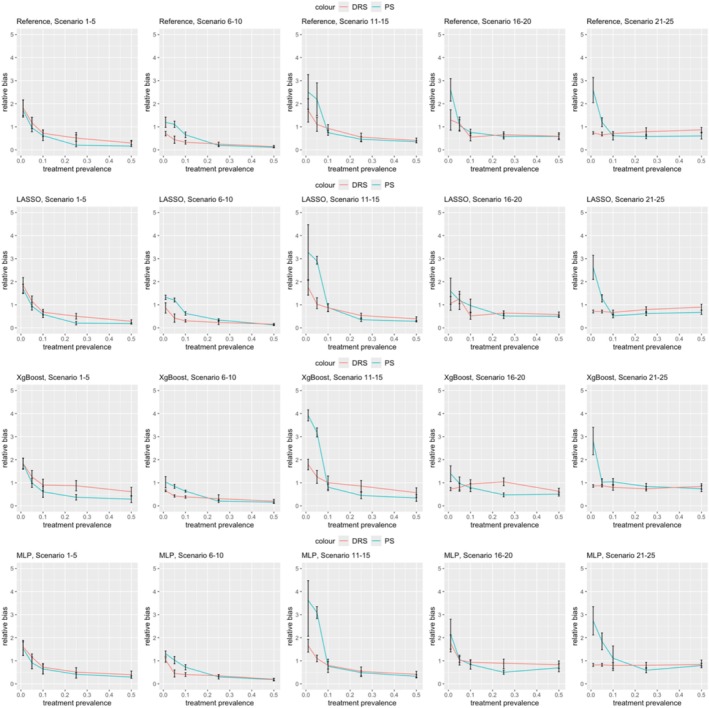
Comparison of bias in treatment effect estimation between PS and DRS across various scenarios. The rows represent methods (from top to bottom: Reference, LASSO, XgBoost, and MLP), while the columns correspond to scenario groups (from left to right: Scenario 1–5, 6–10, 11–15, 16–20, and 21–25).

Figure 1 compares PS and DRS as treatment prevalence changes. As prevalence decreases, PS shows increasing bias, regardless of the ML method, while DRS consistently performs better under these conditions. Particularly in nonlinear data with a prevalence below 0.1, DRS shows significantly lower bias (e.g., in scenario 7, DRS with LASSO had a bias of 0.42 vs. 1.20 for PS). However, this advantage does not hold for linear data; DRS outperforms PS in scenarios with high sample size and low prevalence (Scenarios 6‐8), but PS is superior in linear settings with small samples (Scenarios 1‐5). When the prevalence is moderate to high (0.1‐0.5), PS generally achieves lower bias than DRS, particularly with XgBoost and MLP (Scenarios 11‐20). Brier score loss results can be found in Supplementary Information. The Brier score loss results differed significantly in the last five scenarios due to the lower outcome risk (0.02). Since Brier score loss measures prediction accuracy, lower outcome risk leads to lower Brier score loss as most predictions align with the majority class.

We included a lower outcome risk setting (0.02) when comparing PS and DRS to assess whether our conclusions held with rare events. A detailed comparison in the Supplementary Information shows that ASMD after DRS matching increased and Brier score loss decreased with lower outcome risk across all scenarios, while PS results remained relatively stable when decreasing outcome risk.

**FIGURE 2:**
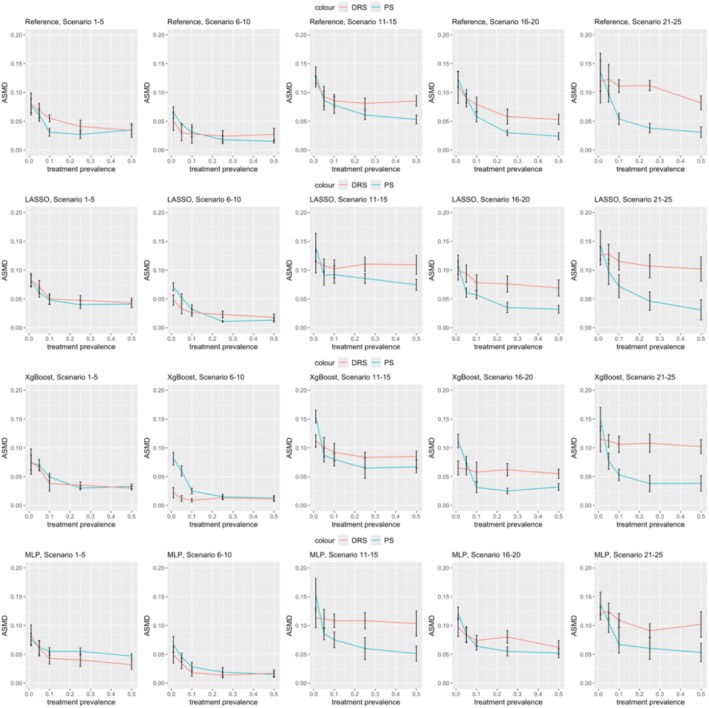
Comparison of ASMD in treatment effect estimation between PS and DRS across various scenarios. The rows represent methods (from top to bottom: Reference, LASSO, XgBoost, and MLP), while the columns correspond to scenario groups (from left to right: Scenarios 1–5, 6–10, 11–15, 16–20, and 21–25).

Figure 2 illustrates the ASMD comparisons between PS and DRS methods. Both methods exhibited an increase in ASMD as treatment prevalence decreased across different ML methods, with the increase being less pronounced when the prevalence ranged between 0.1 and 0.5. In scenarios 1–5, with linear data and a small sample size (500), DRS generally showed higher ASMD than PS. For example, in scenario 1 using LASSO, ASMD post‐DRS matching was 0.0831 (0.0719, 0.0943) compared to 0.0810 (0.0705, 0.0915) post‐PS. Conversely, in scenarios 6–25, ASMD for DRS remained stable, while ASMD for PS increased as prevalence decreased. DRS outperformed or matched PS in ASMD when the prevalence was 0.01. For instance, in scenario 11 using LASSO, ASMD post‐DRS was 0.1143 (0.0951, 0.1335) versus 0.1396 (0.1154, 0.1638) for PS. Overall, DRS was less sensitive to decreasing prevalence in terms of ASMD.

The findings indicate that under our simulation settings, DRS yielded less bias in estimating treatment effects when treatment prevalence was relatively low. Conversely, PS achieved less bias and more balanced covariates in high treatment prevalence scenarios. In high treatment prevalence/low outcome risk scenarios (Scenarios 21–25), ASMD results indicated that PS achieved better balance. However, the similarity in relative bias for treatment effect estimation between PS and DRS may reflect the influence of low outcome risk, where fewer outcome events reduce sensitivity to residual covariate imbalances, resulting in comparable treatment effect estimates.

### 3.2 Effect of Data Complexity

**FIGURE 3:**
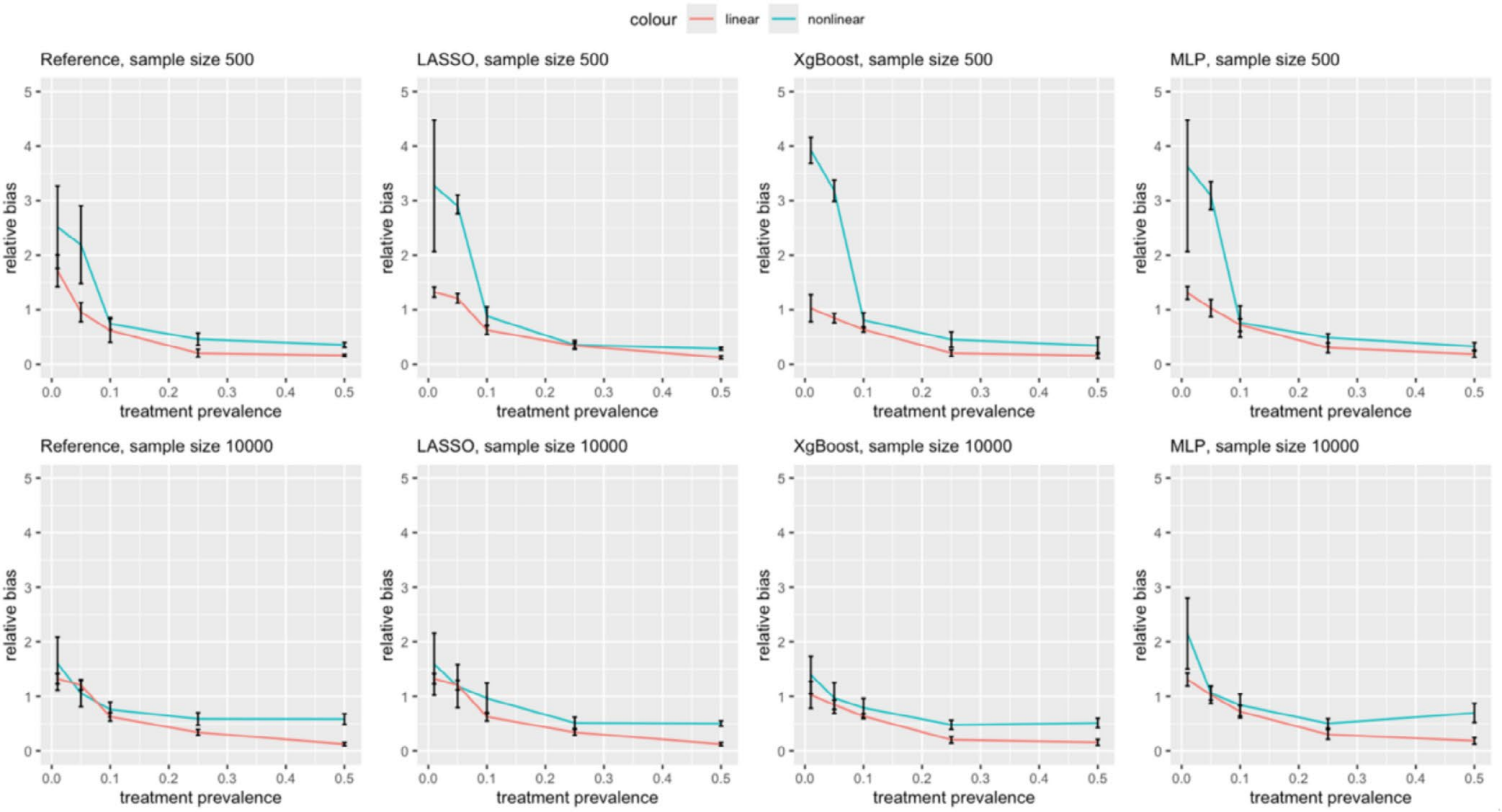
Comparison of treatment effect estimation bias for PS between nonlinear and linear data. From left to right, the methods shown are: Reference, LASSO, XgBoost, and MLP. The first row has a sample size of 500 and the second row has a sample size of 10 000.

**FIGURE 4:**
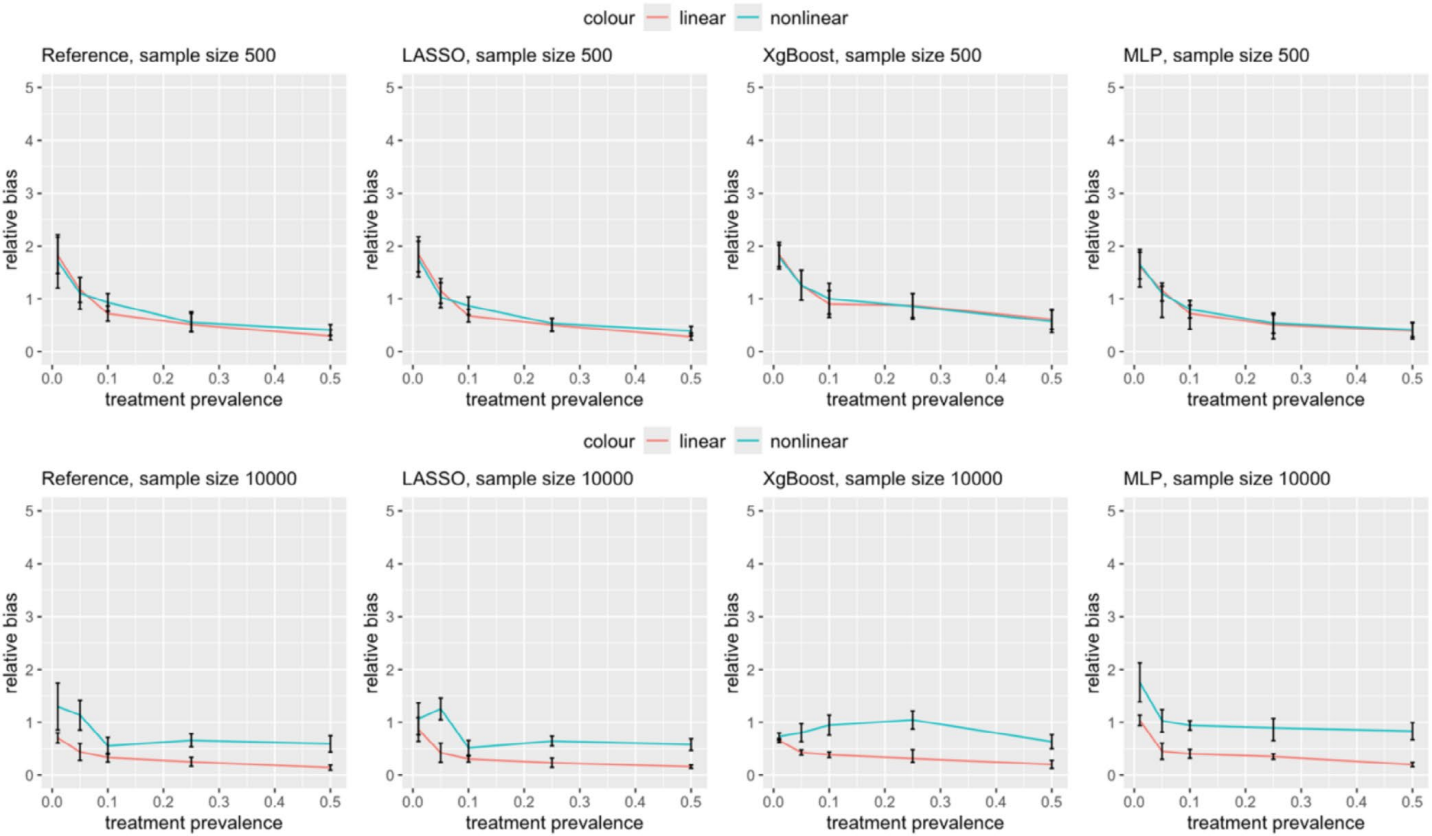
Comparison of treatment effect estimation bias between nonlinear and linear data for DRS. From left to right, the methods shown are: Reference, LASSO, XgBoost, and MLP. The first row has a sample size of 500 and the second row has a sample size of 10 000.

**FIGURE 5:**
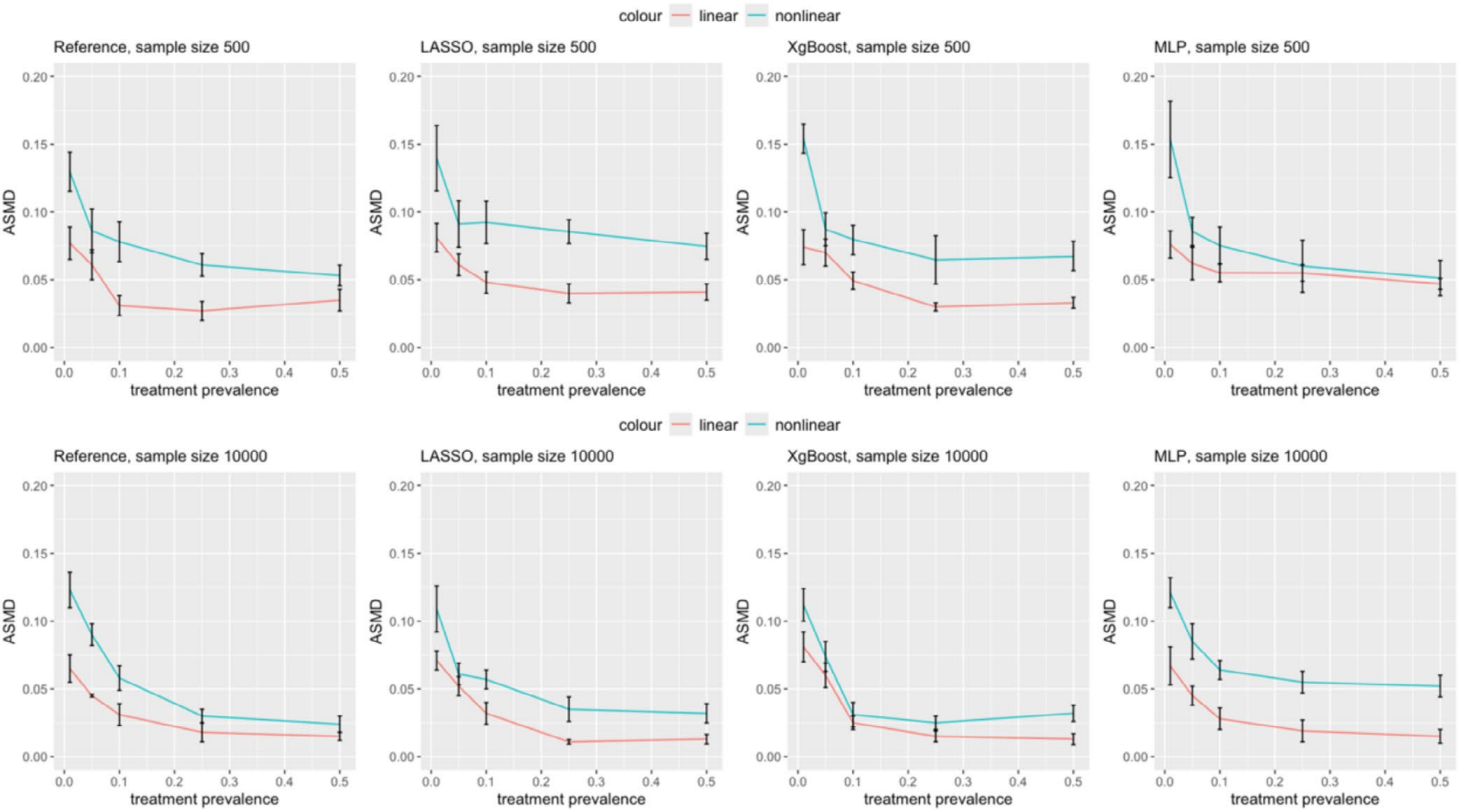
Comparison of ASMD between nonlinear and linear data for PS. From left to right, the methods shown are: Reference, LASSO, XgBoost, and MLP. The first row has a sample size of 500 and the second row has a sample size of 10 000.

**FIGURE 6:**
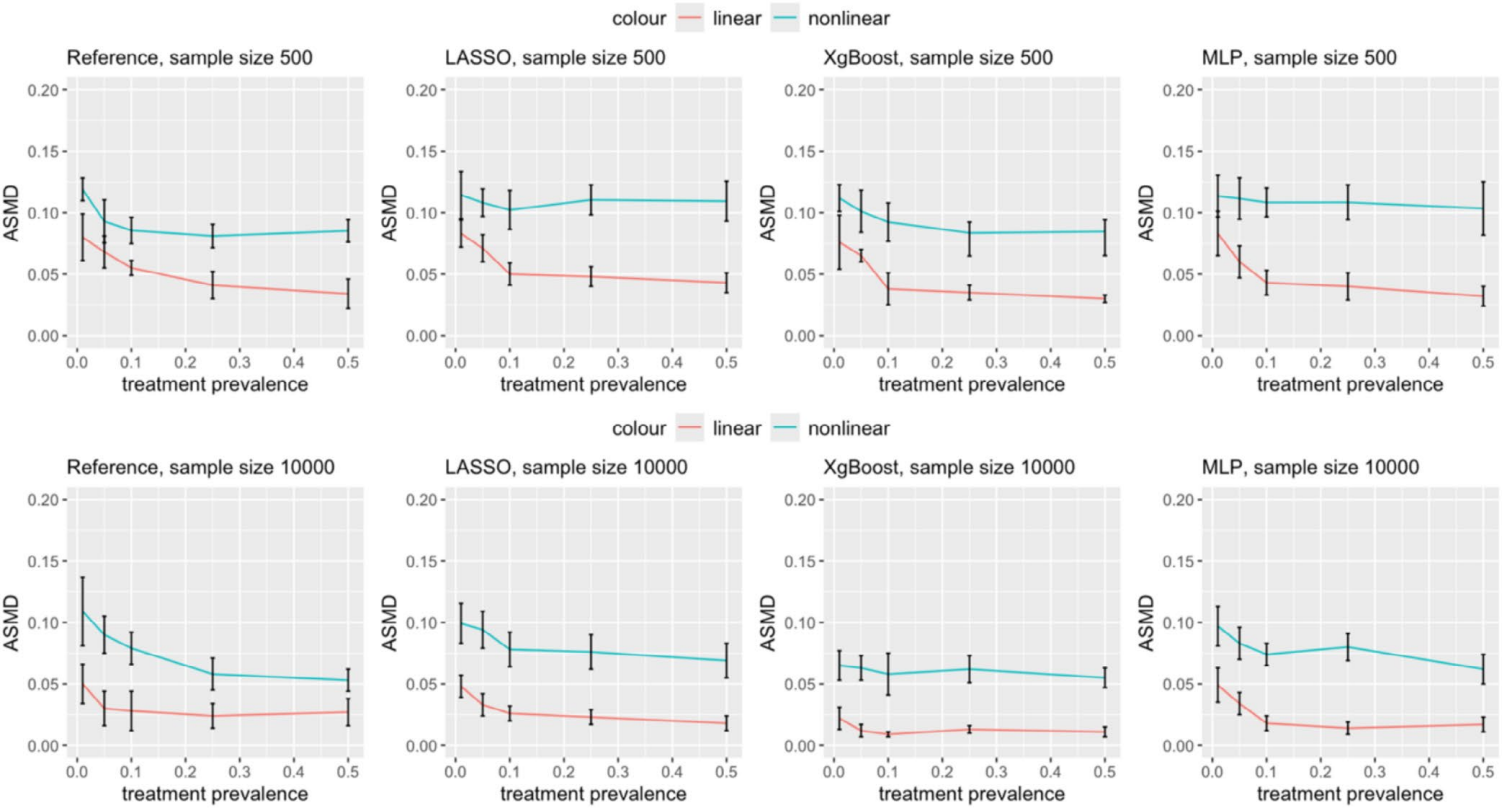
Comparison of ASMD between nonlinear and linear data for DRS. The first row has a sample size of 500 and the second row has a sample size of 10 000.

Figures 3 and 4 illustrate the impact of nonlinearity and nonadditivity on effect estimation for PS and DRS, respectively. Results are shown for sample sizes of 500 (first row) and 10 000 (second row). Across all methods and scenarios, nonlinearity consistently introduced more bias than linearity, with this effect being more significant at lower treatment prevalences. For example, as shown in Figure 3, at a prevalence of 0.01, the relative bias for nonlinear PS using LASSO was 3.27, compared to 1.69 for linear data. Similar trends were observed for ASMD; nonlinear data led to more imbalanced covariates after matching in both PS and DRS, as depicted in Figures 5 and 6.

### 3.3 Effect of Sample Size

**FIGURE 7:**
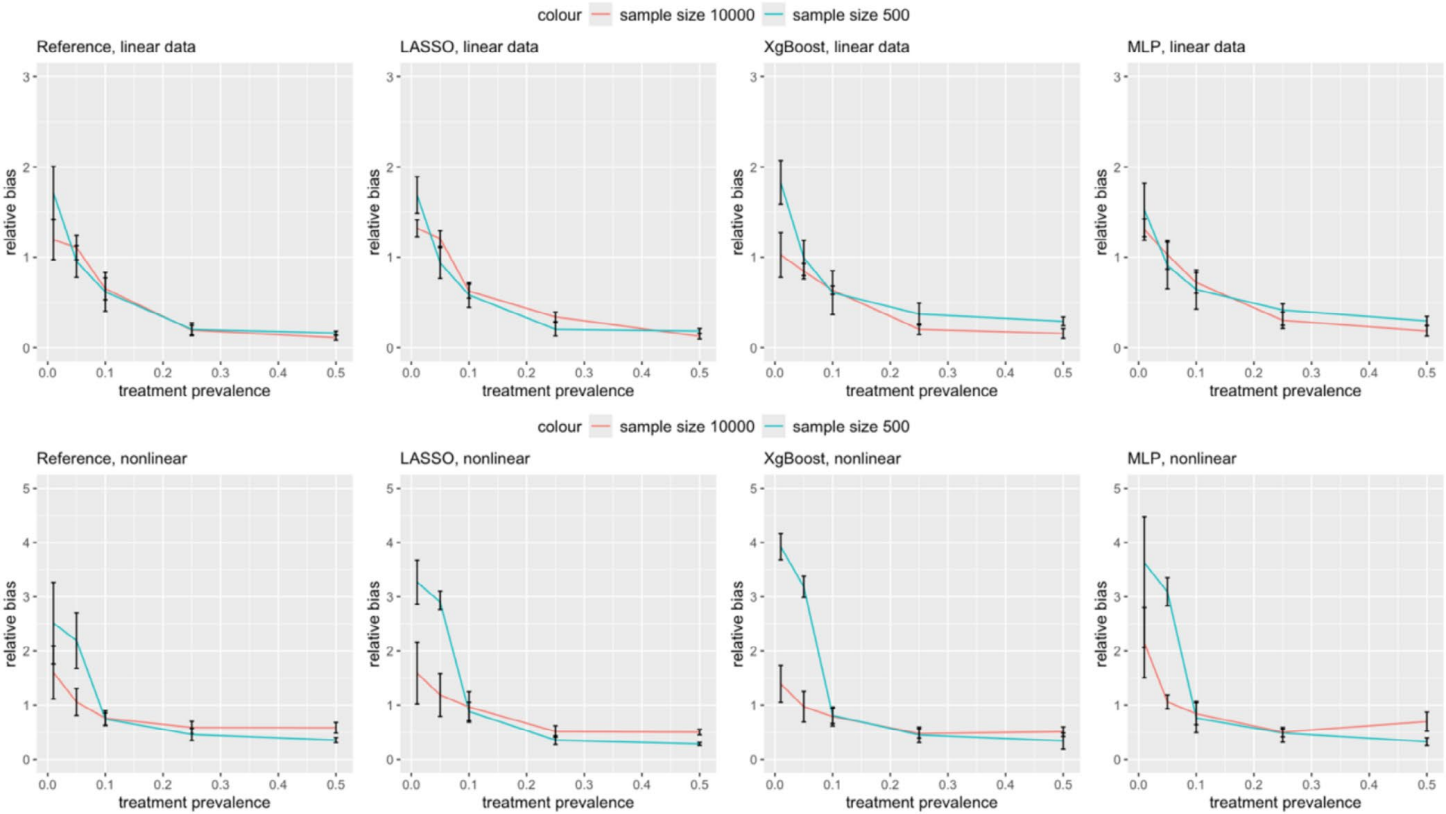
Comparison of treatment effect estimation bias between two sample sizes: 500 and 10 000 for PS. From left to right, the methods shown are: Reference, LASSO, XgBoost, and MLP. The first row shows linear data scenarios and the second row shows nonlinear data scenarios.

**FIGURE 8:**
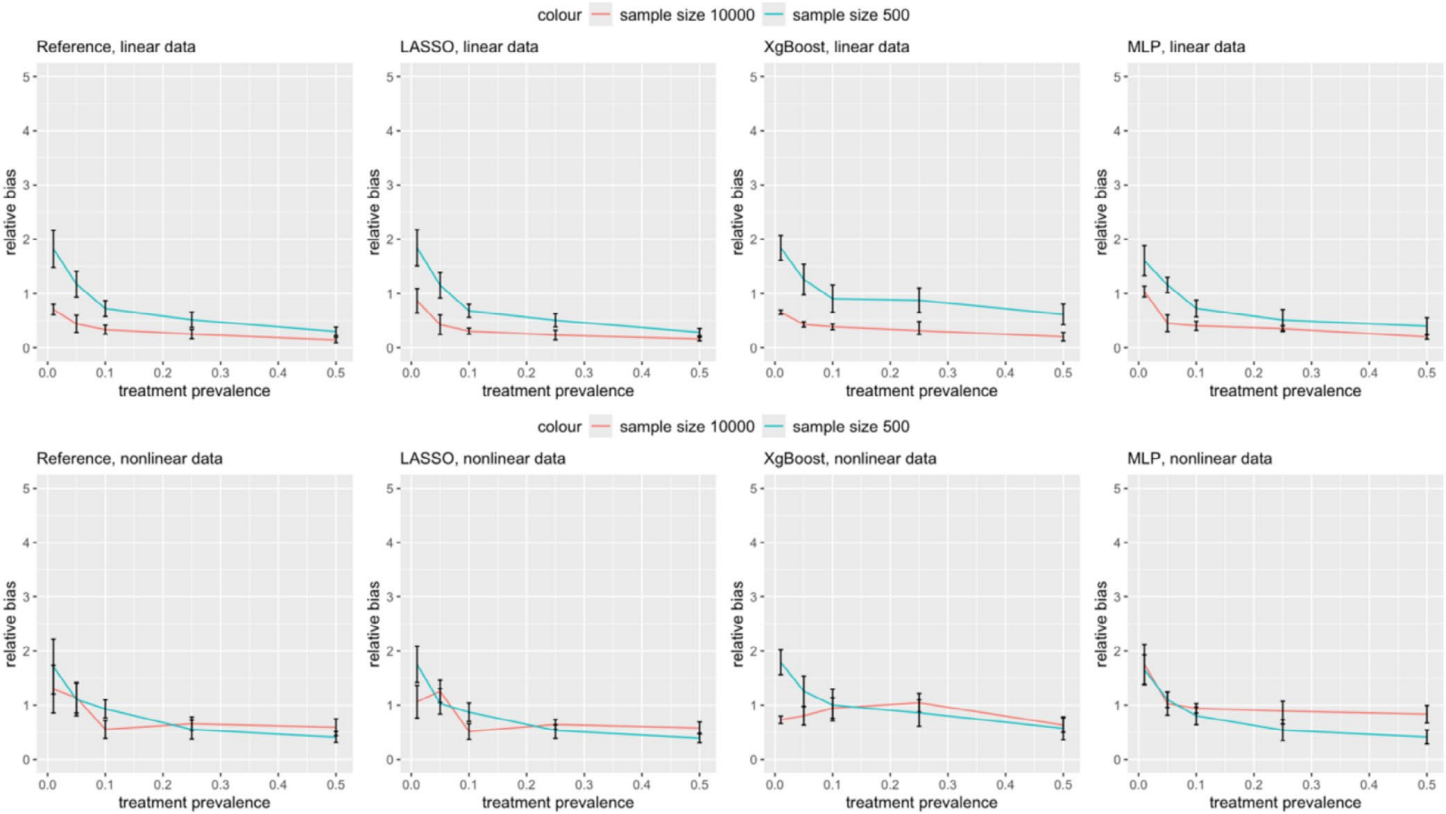
Comparison of treatment effect estimation bias between two sample sizes: 500 and 10 000 for DRS. From left to right, the methods shown are: Reference, LASSO, XgBoost, and MLP. The first row shows linear data scenarios, and the second row shows nonlinear data scenarios.

Figures 7 and 8 show the impact of sample size on treatment effect estimation bias. When the sample size was reduced from 10 000 to 500, the bias remained relatively stable for treatment prevalences between 0.1 and 0.5. However, for prevalences below 0.1, the bias significantly increased for all PS estimation methods.

In linear data scenarios, sample size had a greater effect on DRS bias than on PS bias. At a treatment prevalence of 0.01, reducing the sample size from 10,000 to 500 led to a marked increase in DRS bias across the reference method, XgBoost, LASSO, and MLP, with relative biases rising from 0.70 to 1.82, 0.65 to 1.84, 0.86 to 1.84, and 1.03 to 1.60, respectively. Conversely, in nonlinear data, the reduction in sample size had a more significant impact on PS bias than on DRS at prevalences below 0.1. For example, PS bias increased from 1.60 to 2.51 (reference), 1.59 to 3.27 (XgBoost), 2.35 to 3.62 (LASSO), and 1.39 to 3.92 (MLP) as the sample size decreased.

**FIGURE 9:**
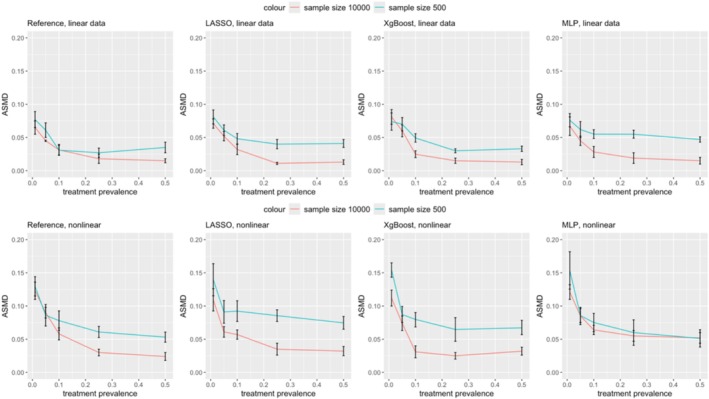
Comparison of ASMD between two sample sizes: 500 and 10 000 for PS. From left to right, the methods shown are: Reference, LASSO, XgBoost, and MLP. The first row shows linear data scenarios and the second row shows nonlinear data scenarios.

**FIGURE 10:**
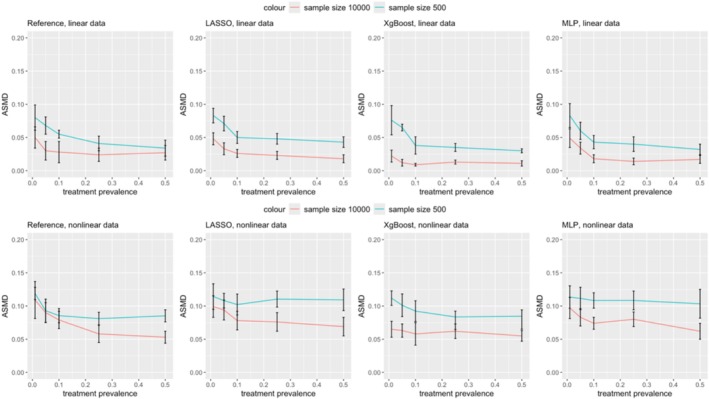
Comparison of ASMD between two sample sizes: 500 and 10 000 for DRS. From left to right, the methods shown are: Reference, LASSO, XgBoost, and MLP. The first row shows linear data scenarios and the second row shows nonlinear data scenarios.

Figures 9 and 10 further illustrate that covariate balance, as measured by ASMD, consistently improved with an increase in sample size from 500 to 10 000.

### 3.4 Comparison of Regression and Machine Learning Methods

**FIGURE 11:**
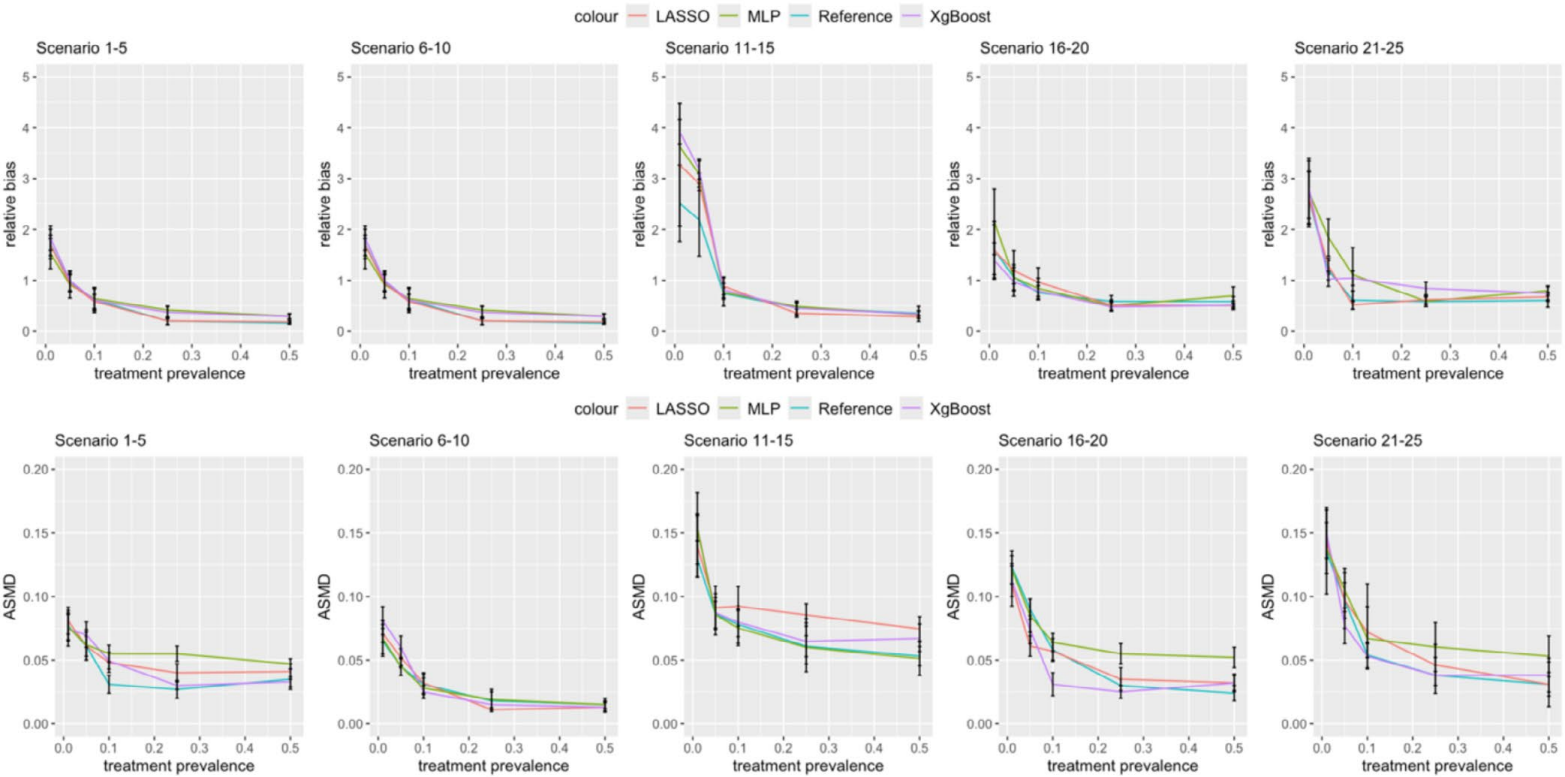
Comparison of different machine learning methods for propensity scores: LASSO, MLP, and XgBoost. From left to right, the columns show Scenarios 1–5, 6–10, 11–15, 16–20, and 21–25. The first row shows relative bias results, while the second row shows ASMD results.

**FIGURE 12:**
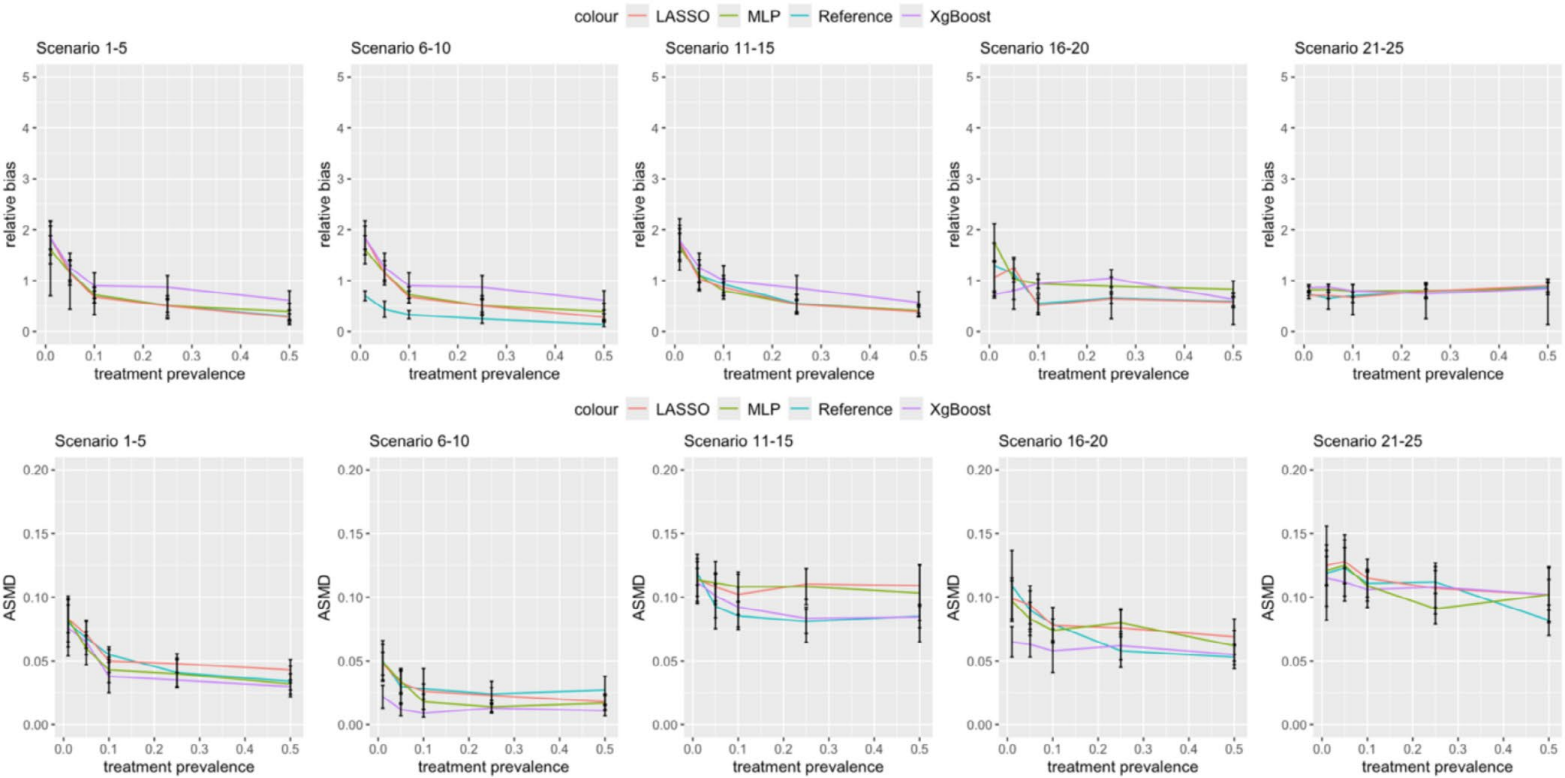
Comparison of different machine learning methods for disease risk scores: LASSO, MLP, and XgBoost. From left to right, the columns show Scenarios 1–5, 6–10, 11–15, 16–20, and 21–25. The first row shows relative bias results, while the second row shows ASMD results.

Figures 11 and 12 compare the bias and ASMD of three ML methods against a reference method for estimating PS and DRS. The first row of each figure shows relative bias, while the second row displays ASMD results.

Across all scenarios, the three ML methods showed similar performance, with overlapping confidence intervals. However, at least one ML method consistently matched or outperformed the reference method, particularly in nonlinear data. XgBoost showed the lowest bias and ASMD when treatment prevalence was below 0.1. For instance, with a prevalence of 0.01, nonlinear data, a sample size of 10 000, and an outcome risk of 0.5, XgBoost estimated PS with a bias of 1.39, compared to 1.60 for the reference method, 1.59 for LASSO, and 2.35 for MLP. In scenario 17 (0.05 prevalence, nonlinear data, 10 000 sample size), XgBoost showed a bias of 0.97, compared to 1.06 for the reference method, 1.19 for LASSO, and 1.06 for MLP.

For linear data scenarios 1–5 (0.1–0.5 prevalence, 500 sample size), LASSO had the lowest bias for both PS and DRS. Additionally, the reference method had the lowest bias for DRS in all linear scenarios 6–10, and the lowest ASMD for PS in linear scenarios 1–5.

## 4 Discussion

We conducted a simulation study to 1) evaluate the performance of DRS and PS for measured confounders adjustment; 2) assess the effects of data complexity and sample size on the performance of PS and DRS; 3) compare the performance of the traditional logistic regression method with pre‐selected confounders to three different ML algorithms.

Regarding the effect of treatment prevalence, for nonlinear data under strong confounding settings, we observed that DRS outperformed PS when treatment prevalence was relatively rare (below 0.1), with lower relative bias and ASMD. This finding aligns with expectations, as models used for PS estimation may fail to capture nonlinearity and nonadditivity when the target variable (treatment) is imbalanced. We also found that when treatment prevalence was between common and high (0.1‐0.5), PS resulted in less bias and more balanced covariates compared to DRS. Previous research by Xu et al. [6] compared PS and DRS using Type I error and empirical power, which are additional metrics that could be used in simulation studies. They found that for 1:1 matching, PS outperformed DRS, as PS had lower Type I errors. Our results were consistent with Xu et al., showing that PS outperformed DRS when treatment prevalence was between common and high (0.1‐0.5) in scenarios 1‐5, 9, 10, 13‐15, 19, 20, and 25. However, their study did not include scenarios where DRS might be advantageous over PS, such as when both treatment prevalence (below 0.1) and outcome risk (0.02) were relatively rare, as demonstrated by our results. This discrepancy could be due to differences in simulation settings, as confounding strength with treatment and outcome was randomly generated in each of our simulation rounds. Additionally, the difference in evaluation metrics used may have contributed. We also observed that the ASMD generated by DRS was less affected by the decrease in treatment prevalence compared to that of PS. Importantly, our study controlled for treatment prevalence and outcome risk, which inherently affected overlap in PS and DRS distributions, and therefore we did not explicitly control for overlap. We assessed balance using ASMD after matching, which directly reflects comparability in the matched cohort. While our findings provide insights into the relative performance of PS and DRS, future studies could explore the effects of explicitly controlling for overlap on method performance.

Sample size also affected our estimation bias. With only 500 observations, 50 confounders, and the nonlinear data setting, our data suggested that PS resulted in significant bias when the outcome risk was 0.02 and treatment prevalence was below 0.1. This can be explained by the number of events per variable being too low, as we had 50 confounders, and when treatment prevalence was below 0.1, we had fewer than one event per confounder. As previous research has demonstrated, even 10 events per variable may not be sufficient [21]. Previous research comparing DRS and PS [5] also demonstrated that for logistic regression, reducing the number of events per covariate increased bias for both PS and DRS. We observed that for linear data, sample size affected DRS more than PS. This is expected as modeling the DRS introduces additional complexity, requiring accurate modeling of the relationship between treatment and outcome. Therefore, decreasing the sample size under these scenarios could make it more difficult for DRS than PS. However, after adding nonlinearity and nonadditivity to the data, we observed that a small sample size with decreasing treatment prevalence had a greater effect on PS than DRS. Further studies into this area could be explored in future research.

For the traditional logistic regression method and three ML methods we tested, reference method, LASSO, XgBoost, and MLP, all ML had similar performance when we had complicated nonlinear data settings. When treatment prevalence was between common and high (0.1‐0.5), we observed that at least one ML method outperformed the reference method. LASSO under many linear scenarios outperformed the reference method, likely due to its ability to perform variable selection and mitigate overfitting in small sample sizes. Whereas both LASSO and XgBoost outperformed the reference method under some nonlinear scenarios, XgBoost demonstrated superior performance in nonlinear settings with large sample sizes and low treatment prevalence, likely due to its ability to capture complex interactions and nonlinear relationships while effectively leveraging large datasets. A previous review [10] found that the boosting method was the most promising ML method for PS analysis, which was consistent with our observation that XgBoost as a boosting method outperformed other methods under many scenarios. High‐dimensional PS [13] is also a popular method to estimate PS. It includes a process of ranking covariates by their prevalence and univariate association with the outcome and/or the treatment, and it requires a specific pre‐selected number of covariates. However, our approach was to allow ML to self‐select among potentially large numbers of confounders. While our study applied the same modeling approach for PS and DRS to ensure a fair comparison, we acknowledge that further improvements in PS estimation could be achieved by incorporating additional transformations such as logarithmic, sine, or interaction terms. However, given our data‐driven approach, manually optimizing PS and DRS for balance in each scenario would reduce comparability across methods and introduce additional complexity. Future studies could explore more tailored PS and DRS modeling strategies to assess their impact on balance and bias. Additional ML methods such as outcome adaptive LASSO [22] and highly adaptive LASSO [23] have also been illustrated to have competitive performance compared with other popular ML techniques like gradient boosted machine [23]; however, they were mostly compared on small scale data for computational reasons. It could be beneficial to further explore them in future research.

While ASMD results generally align with relative bias results, discrepancies were observed in scenarios with low outcome risk. For instance, in Scenarios 21–25 (high treatment prevalence/low outcome risk), PS achieved better ASMD than DRS, but the relative bias for treatment effect estimation was similar. This may be explained by the reduced sensitivity of bias to residual covariate imbalances when outcome events are sparse. Additionally, prior research has indicated that covariate balance measures like ASMD may not always correlate perfectly with bias reduction, particularly when covariates differ in their relative importance to treatment and outcome [24]. These findings underscore the complexity of interpreting balance measures in the context of treatment effect estimation. Our findings also suggest that ML‐based DRS estimation can have better relative bias under some low treatment prevalence scenarios; it does not achieve comparable covariate balance to PS in scenarios with moderate‐to‐high treatment prevalence. PS generally achieved better covariate balance than DRS, even when both methods resulted in similar treatment effect estimates. This suggests that while ML‐based DRS can still yield reasonable effect estimates, its use in these settings may require careful consideration of residual imbalance.

All scenarios tested in this study were based on simulated data. While simulation studies are important tools for statistical analysis, particularly for studying causal effects, we acknowledge the limitations inherent in using synthetic data. Expanding the scenarios to include an increased number of risk factors, instrumental variables, nonlinearities, and nonadditivities could provide a deeper understanding of what affects the estimation of PS and DRS. However, such expansions were beyond the scope of this study. Future research could be extended to include more simulated data, plasmode simulations, and real‐world data to further validate and enhance the findings [25].

## 5 Conclusion

We compared the performance of the less‐known DRS with the widely used PS for estimating treatment effects in the presence of strong confounding and large sample sizes. Both PS and DRS were computed using logistic regression and three well‐established ML methods. Our data suggest that DRS could be a preferable method compared to PS when treatment prevalence is below 0.1, particularly under strong confounding and nonlinear data scenarios. PS demonstrated competitive performance compared to DRS when treatment prevalence ranged between 0.1 and 0.5. The ASMD of covariates generated by PS increased more significantly with decreasing treatment prevalence compared to DRS. Sample size also played a crucial role, as a small sample size may lead to inaccurate estimations for both DRS and PS under strong confounding conditions. Among the methods tested, XgBoost and LASSO can outperform traditional logistic regression, with XgBoost often excelling in nonlinear scenarios and LASSO in linear scenarios.

### 5.1 Plain Language Summary

This study was motivated by the surge of COVID‐19 treatments in 2020, which had low treatment prevalence and high outcome risk. We conducted a simulation to compare the effectiveness of two statistical methods—DRS and PS—in estimating treatment effects across various scenarios. These scenarios varied in treatment prevalence, data complexity, and sample size. We used traditional regression and three ML methods—LASSO, MLP, and XgBoost—to estimate PS and DRS. Our findings showed that when treatment prevalence was low (less than 0.1), DRS provided lower bias compared to PS, particularly in complex, nonlinear data. However, PS performed better in scenarios with higher treatment prevalence (0.1–0.5). Among the ML methods, LASSO and XgBoost can outperform traditional regression under some nonlinear scenarios. Overall, the study highlights the importance of choosing the right method based on data characteristics, particularly in situations with strong confounding and low treatment prevalence.

## Conflicts of Interest

Prof. Daniel Prieto‐Alhambra's research group has received grant support from Amgen, Chesi‐Taylor, Novartis and UCB Biopharma. His department has received advisory or consultancy fees from Amgen, Astellas, AstraZeneca, Johnson and Johnson, and UCB Biopharma and fees for speaker services from Amgen and UCB Biopharma. Janssen, on behalf of IMI‐funded EHDEN and EMIF consortiums and Synapse Management Partners, has supported training programmes organised by DPA's department and open for external participants organised by his department, all unrelated to the submitted work.

## Supporting information


Data S1.
