# Supplementary material for: Use of Machine Learning to Compare Disease Risk Scores and Propensity Scores Across Complex Confounding Scenarios: A Simulation Study
Source: Pharmacoepidemiol Drug Saf. 2025 Jun 2;34(6):e70165. doi: 10.1002/pds.70165 (PMC12130674; doi:10.1002/pds.70165)
Supplement: Supplementary file 1 — Data S1. [file PDS-34-e70165-s001.docx]

Code availability: All code for this paper can be found in [https://github.com/MimimimiGuo/PS](https://github.com/MimimimiGuo/PS_ML)_[ML.](https://github.com/MimimimiGuo/PS_ML)

Figure S1: Simulation flowchart: demonstration of how linear and nonlinear data structures were simulated.


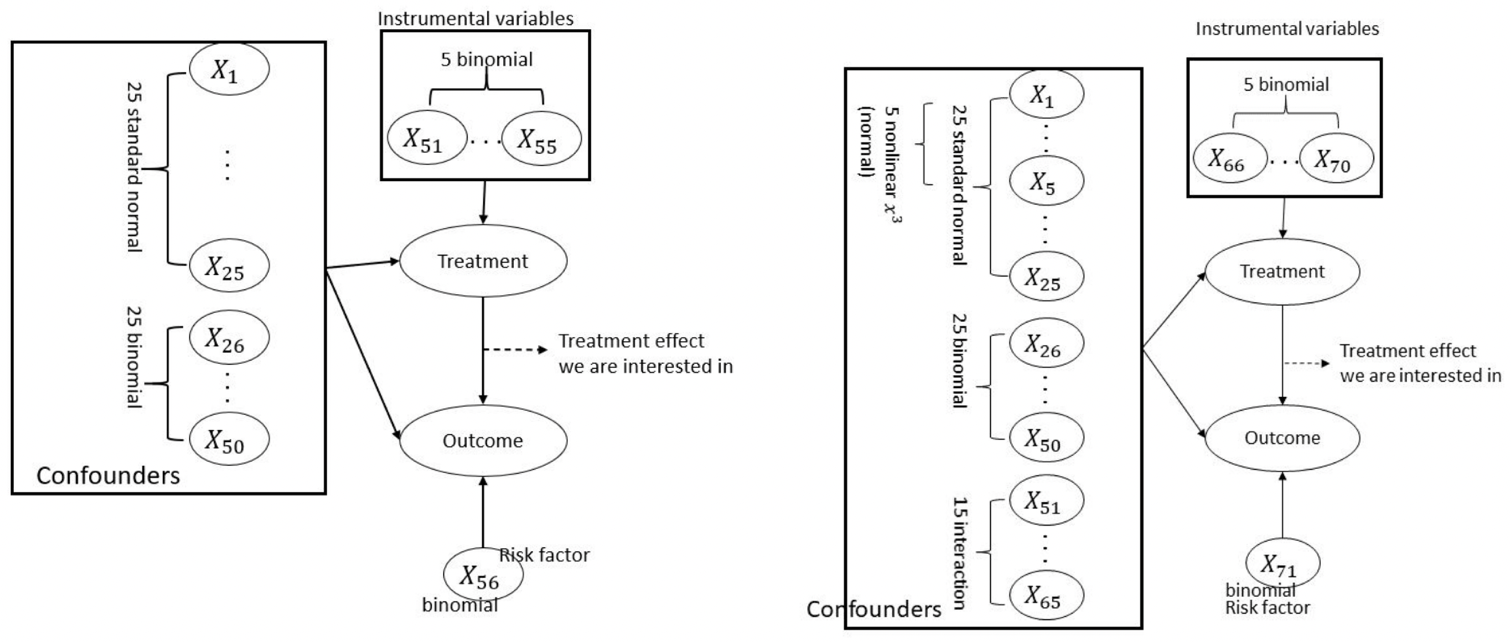


Figure S2: Brier score loss for all scenarios for reference, LASSO, XgBoost and MLP methods
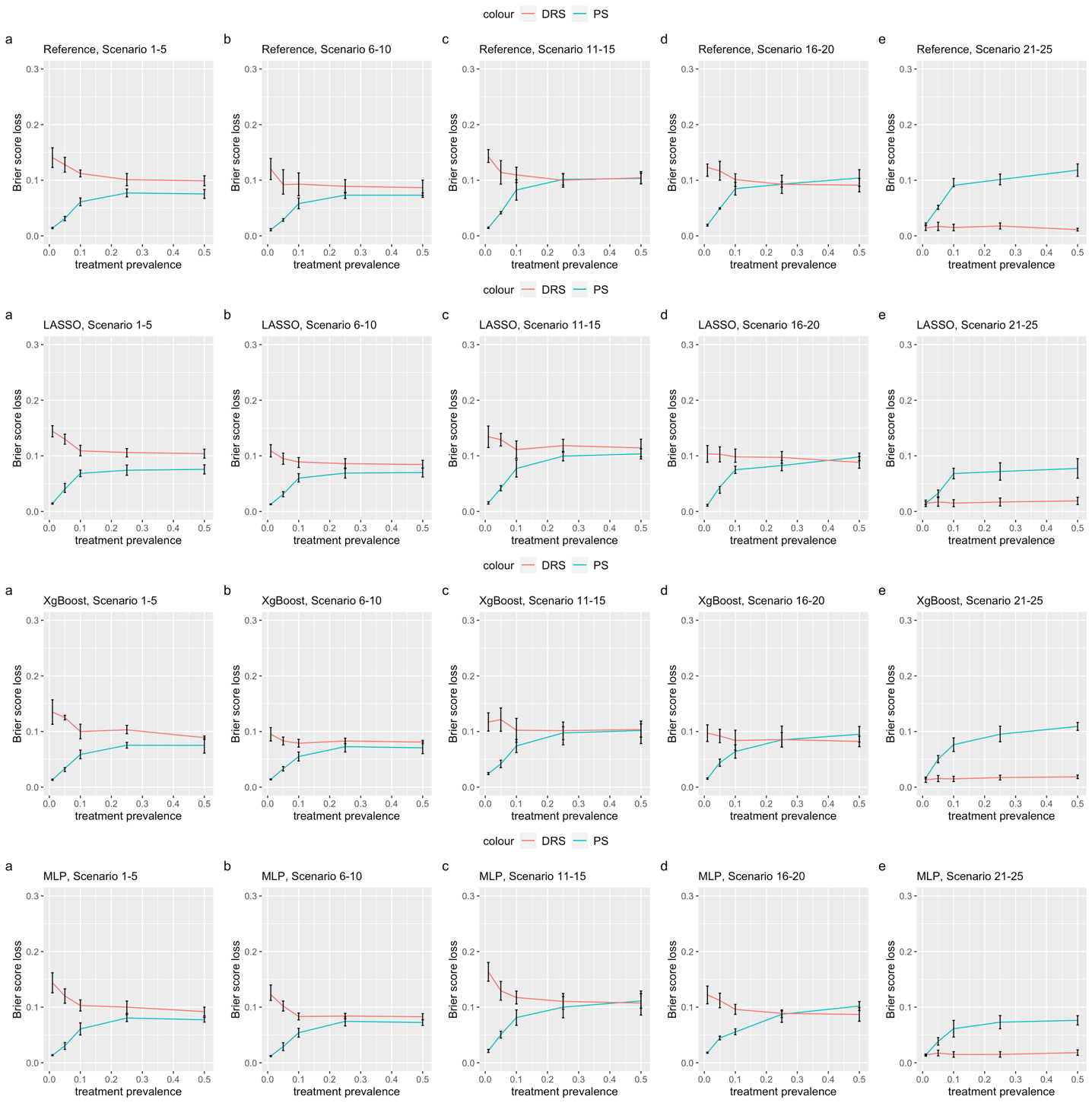


Figure S3: outcome risk comparison 0.02 vs 0.5 for disease risk scores, for LASSO, MLP, XgBoost and reference method.
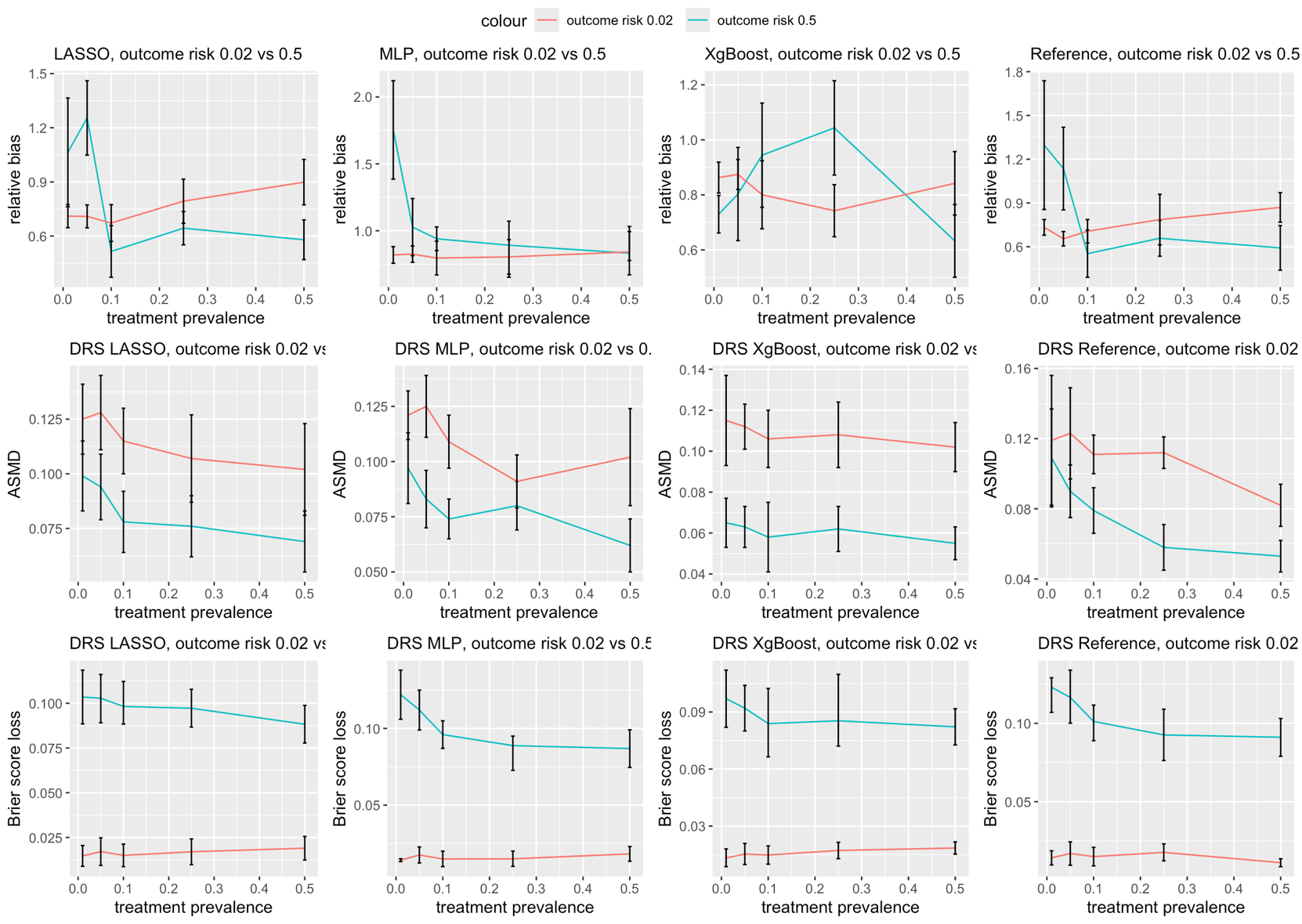


Figure S4: outcome risk comparisons 0.02 vs 0.5 for propensity scores, for LASSO, MLP, XgBoost and reference method.
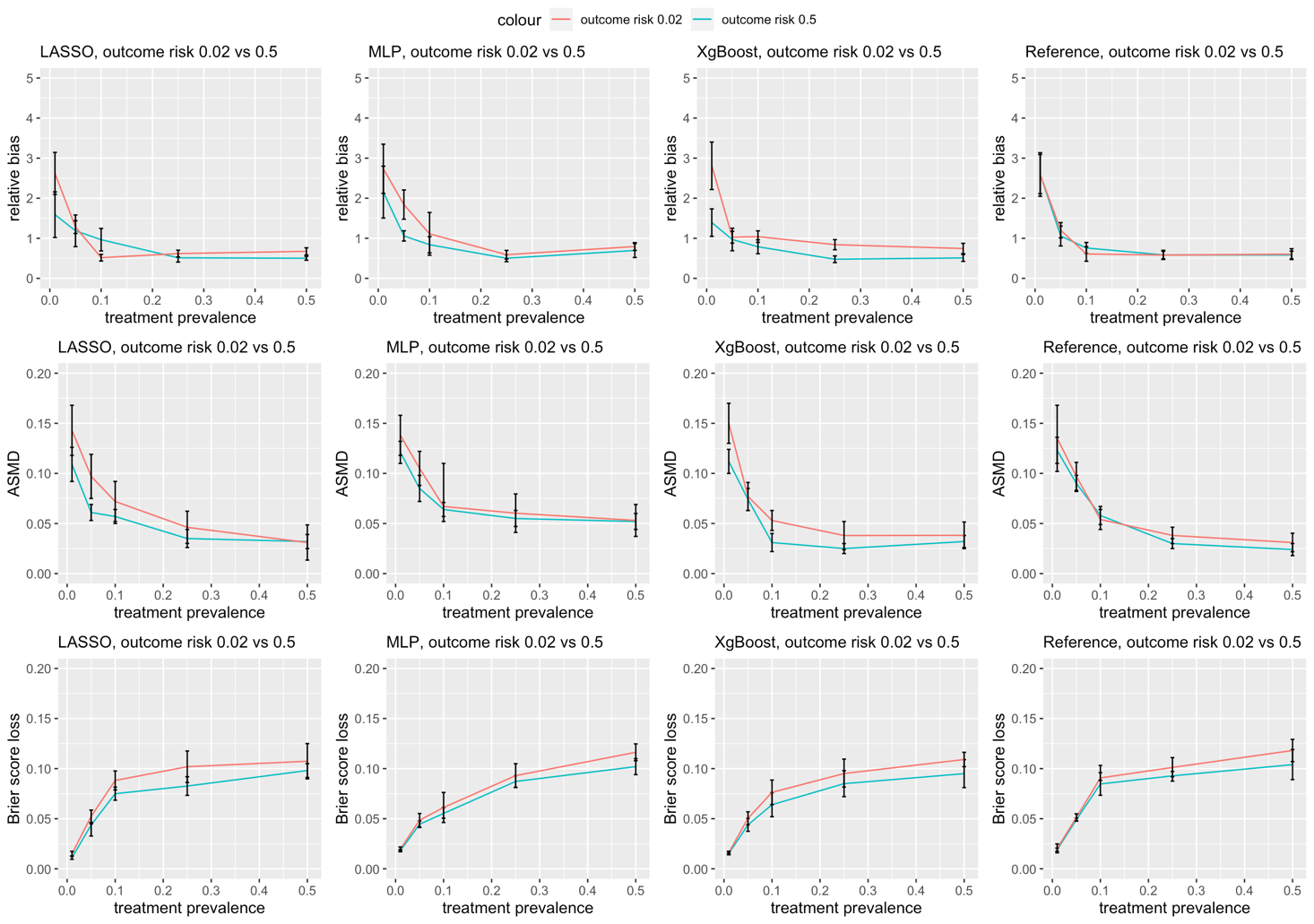


Table S1: LASSO hyperparameters tuned

| Hyperparameter | Range |
| --- | --- |
| C (alpha) | 0.01 to 0.31 (step 0.02) |
| solver | ['liblinear', 'saga'] |

Table S2: MLP hyperparameters tuned

| Hyperparameter | Range |
| --- | --- |
| batch_size | [10, 32, 64] |
| epochs | [10, 100, 1000] |
| optimizer | ['adam', 'rmsprop', 'SGD'] |
| kernel | ['random_normal', 'random_uniform', 'truncated_normal'] |
| units | [8, 32, 64, 128] |
| hidden_layers | [2, 3, 5, 7] |
| activation | ['tanh', 'sigmoid', 'relu', 'selu'] |

Table S3: XgBoost hyperparameters tuned

| Hyperparameter | Range |
| --- | --- |
| n_estimators | [100, 300, 600, 1000] |
| min_child_weight | [1, 10, 50] |
| gamma | [0.5, 2, None] |
| subsample | [0.6, 0.8, 1.0] |
| learning_rate | [0.02, 0.1, 0.2, 0.5] |
| max_depth | [3, 5, 7, 12] |

Hyperparameters tuned are shown in Table S1, S2 and S3. All modelling were done in Python using xgboost package (<https://xgboost.readthedocs.io/en/stable/python/>), keras package (<https://pypi.org/project/keras/>) and scikit-learn package (https://scikit-learn.org/stable/).
